# Complement-dependent outer membrane perturbation sensitizes Gram-negative bacteria to Gram-positive specific antibiotics

**DOI:** 10.1038/s41598-019-38577-9

**Published:** 2019-02-28

**Authors:** D. A. C. Heesterbeek, N. I. Martin, A. Velthuizen, M. Duijst, M. Ruyken, R. Wubbolts, S. H. M. Rooijakkers, B. W. Bardoel

**Affiliations:** 1 Medical Microbiology, University Medical Center Utrecht, Utrecht University, Utrecht, Netherlands; 2 Institute of Biology Leiden, Leiden University, Leiden, Netherlands; 3 Department of Biochemistry and Cell Biology, Utrecht University, Utrecht, Netherlands

**Keywords:** Complement cascade, Antimicrobial resistance

## Abstract

Gram-negative bacteria are refractory to the action of many antibiotics due to their impermeable outer membrane. An important player of the immune system is the complement system, a protein network in serum that directly kills Gram-negative bacteria through pore-formation by the Membrane Attack Complexes (MAC). We here show that the MAC rapidly perforates the outer membrane but that inner membrane damage, which is essential for killing, is relatively slow. Importantly, we demonstrate that MAC-induced outer membrane damage sensitizes Gram-negative bacteria to otherwise ineffective, Gram-positive-specific, antimicrobials. Synergy between serum and nisin was observed for 22 out of 53 tested Gram-negative clinical isolates and for multi-drug resistant (MDR) blood isolates. The *in vivo* relevance of this process is further highlighted by the fact that blood sensitizes a MDR *K. pneumoniae* strain to vancomycin. Altogether, these data imply that antibiotics that are considered ineffective to treat infections with Gram-negatives may have different functional outcomes in patients, due to the presence of the complement system.

## Introduction

Gram-negative bacteria form a major threat for human health in community and hospital settings. The increasing rates of multi-drug resistant (MDR) Gram-negative bacteria form a major obstacle for successful treatment of infections^1–3^. Drug-resistant forms of pathogenic *Klebsiella pneumoniae, Acinetobacter baumannii, Pseudomonas aeruginosa* and *Enterobacter* species are considered the most problematic since there are limited therapeutic options left for these infections. The number of newly developed antibiotics is historically low and new antimicrobial strategies are desired to prevent further rise of untreatable infections with MDR bacteria^4,5^. This is supported by the World Health Organization, which strongly prioritizes the development of new “out of the box” strategies to treat infections with MDR bacteria^6^. Development of novel antibiotics against Gram-negative bacteria is hampered by the bacterial outer membrane (OM) that functions as a physical barrier to most antibiotics^7^. Whereas antibiotics targeting the peptidoglycan (PG) layer and the inner membrane (IM) can generally access their targets on Gram-positive bacteria, the OM of Gram-negative bacteria (comprising phospholipids and lipopolysaccharides (LPS)) forms a physical barrier for many antibiotics^7–10^. Outer membrane permeability for antibacterial compounds largely depends on the size, polarity and lipophilicity of these compounds, which influence the efficiency with which antimicrobials diffuse over the outer membrane via porins^11,12^. This limits the number of antibiotics suitable for treating infections with Gram-negative bacteria.

The action of antibiotics may be influenced *in vivo* by a patient’s immune system that has mechanisms to affect bacterial membrane permeability. In previous studies, synergy was observed between serum and antibiotics for Gram-negative bacteria, however the mechanism of synergy remains unclear^13,14^. We hypothesized that complement proteins, present in the blood and most bodily fluids, are responsible for these synergistic effects. Upon contact with invading bacteria, a step-wise activation process results in the rapid decoration of bacteria with complement proteins and insertion of Membrane Attack Complex (MAC) pores into bacterial membranes. This can lead to direct killing of Gram-negative, but not Gram-positive bacteria. These evolutionary conserved MACs are large, multi-molecular ring-structured pores with an inner diameter of ~10 nm^15^, that consist of 5 different proteins (C5b, C6, C7, C8 and 18 copies of C9)^16^. Recently we observed that when the MAC properly forms pores in the outer membrane of *E. coli*, this triggers inner membrane damage and killing^17^. Here we show that MAC-dependent outer membrane damage is much faster and more efficient than loss of inner membrane integrity and killing, which is a relatively inefficient process. However, the fast perforation of the outer membrane by the complement system allows naturally impermeable antibiotics to reach their target sites and kill Gram-negative bacteria. We observed that the Gram-positive specific antibiotics nisin and vancomycin can be active against Gram-negatives in the presence of complement. We show for the first time that complement sensitizes a broad range of clinical isolates (including MDR strains) to antibiotics that are currently considered ineffective for treatment of Gram-negative bacteria.

## Results

### Complement-mediated outer membrane damage is more efficient than inner membrane damage

**Figure 1:**
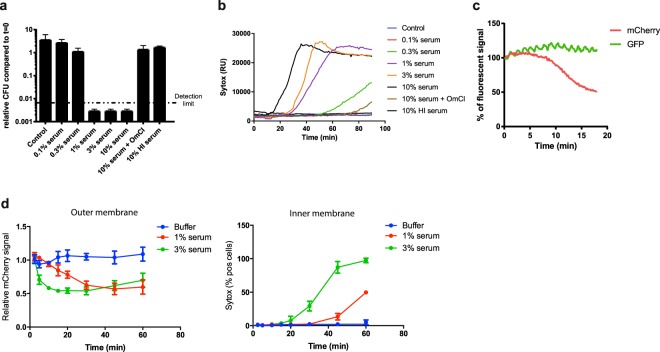
Complement-dependent outer membrane permeabilization is more efficient than inner membrane destabilization. (**a**) *E. coli* was exposed to a serum concentration range for 60 minutes at 37 °C. Viability was determined using colony counting before and after exposure to buffer, different concentrations of serum, heat inactivated (HI) serum or 10% serum + 20 µg/ml MAC inhibitor OmCI. Viability was calculated relative to the CFU/ml of untreated samples at t = 0. (**b**) Dynamic analysis of IM destabilization of *E. coli* following incubation with 0-0.1-0.3-1-3-10% serum at 37 °C. Sytox intensity was measured every minute for 90 minutes in a microplate fluorometer. Complement specificity was confirmed using 20 µg/ml OmCI and 10% HI serum. (**c**) Dynamic analysis of outer membrane integrity of MG1655-pPerimCh upon exposure to 3% serum at RT. mCherry and GFP intensity were measured in time by flow cytometry. (**d**) Outer membrane (mCherry, left) and inner membrane (% Sytox positive, right) damage of MG1655-pPerimCh after exposure to buffer, 1% or 3% serum. mCherry and Sytox intensities were measured at different time-points using flow cytometry. (**b**,**c**) A representative graph of at least three independent experiments is shown. (**a**,**d**) Data represent mean ± SD of three individual experiments.

In order to study how the MAC kills bacteria, we used a flow cytometry based assay to measure serum-induced outer and inner membrane damage in *E. coli*^17^. In short, we generated an *E. coli* strain that expresses mCherry in the periplasm and GFP in the cytoplasm (Suppl. Fig. 1a,b). Upon exposure to human serum, the leakage of fluorescent proteins from the different cell compartments was measured by flow cytometry. In addition, incubations were performed in the presence of the small, IM impermeable DNA dye Sytox that becomes fluorescent upon binding to DNA^18^ (Suppl. Fig. 1c). Although we recently found that killing of Gram-negative bacteria by human complement requires permeabilization of both membranes, the dynamics and efficiency of this process remained unclear. To study how fast serum perturbs the inner membrane and thus triggers killing of *E. coli*, we incubated bacteria with a concentration range of serum, measured inner membrane damage in a multi-well plate reader and determined bacterial viability. Although 1, 3 and 10% serum efficiently killed *E. coli* within an hour (Fig. 1a), it took 20 to 60 minutes to actually damage the inner membrane and kill the bacteria (depending on the serum concentration) (Fig. 1b). Sytox influx and bacterial killing was fully blocked by complement inhibitor OmCI, confirming that IM damage was caused specifically by the MAC (Fig. 1a,b)^19^. Then we measured the dynamics of MAC-dependent outer membrane damage using mCherry/GFP bacteria. mCherry release was measured in real-time by flow cytometry. In contrast to the slow Sytox influx, we found a rapid decrease of the periplasmic mCherry signal after addition of serum (Fig. 1c and Suppl. Fig. 1d). OM permeabilization started after around 7 min and maximal mCherry leakage was reached after 15 minutes at room temperature, when bacteria still had an intact inner membrane (Fig. 1b,c). Serum did not affect the levels of cytosolic GFP, again indicating that only the OM is damaged within this time frame (Fig. 1c). The mCherry release specifically depended on MAC components C8 and C9 (Suppl. Fig. 1e). To directly compare the dynamics of outer and inner membrane damage on a single cell level, we incubated mCherry/GFP *E. coli* with serum in the presence of Sytox and measured mCherry and Sytox intensity in time by flow cytometry. We observed that the outer membrane is damaged faster and more efficiently than the inner membrane (Fig. 1d). Whereas 3% serum efficiently damaged the outer membrane within 10 minutes, influx of Sytox started only after 30 minutes and reached its maximum after more than 40 minutes at room temperature. In addition, while 1% serum fully damaged the bacterial outer membrane within 30 minutes, inner membrane damage was much less efficient (Fig. 1d).

In summary, we show that MAC-dependent perturbation of the OM precedes IM destabilization. Whereas the MAC efficiently permeabilizes the OM of Gram-negative bacteria, longer incubation times or higher serum concentrations are necessary for IM perturbation by complement alone.

### The MAC forms pores in the outer membrane that allow nisin to reach the inner membrane

**Figure 2:**
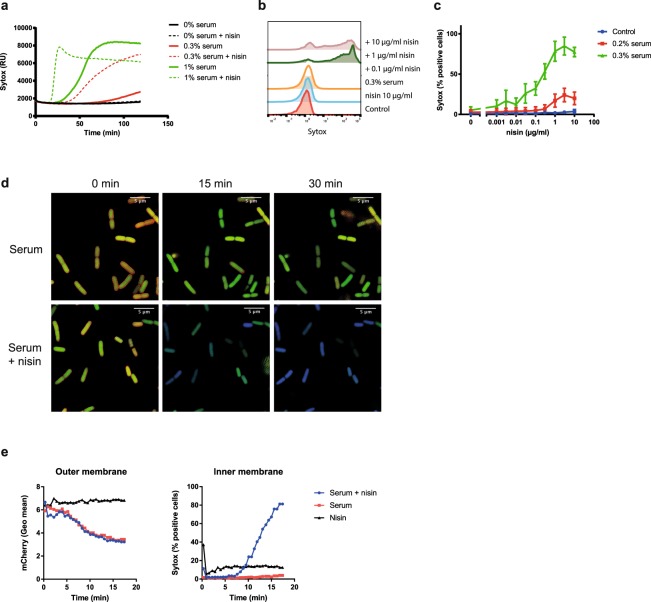
The MAC forms pores in the outer membrane that allow influx of nisin. (**a**) Inner membrane damage (Sytox intensity) of *E. coli* MG1655 treated with buffer, 0.3% or 1% serum in the presence or absence of 10 µg/ml nisin at 37 °C. Sytox intensity was measured in a multi-well plate reader. (**b**) Flow cytometric analysis of Sytox green signal of bacteria incubated with different nisin concentrations in the presence or absence of serum. (**c**) Flow cytometric analysis of inner membrane damage (% Sytox positive) of MG1655 treated with 0-0.2-0.3% serum in the presence of various nisin concentrations for 60 min at 37 °C. (**d**) Reconstructed SIM image of MG1655**-**pPerimCh incubated with 3% serum in presence and absence of 3 µg/ml nisin for 0, 15 or 30 minutes at RT. Sytox blue, GFP and mCherry were measured using the 405, 488 and 561 nm lasers. (**e**) Flow cytometry time-lapse experiments of outer membrane (left) and inner membrane (right) integrity of MG1655-pPerimCh exposed to 3% serum in the presence or absence of 3 µg/ml nisin at RT. (**a**,**b**,**e**) Representative graph of at least three independent experiments is shown. (**c**) Data represent mean ± SD of three individual experiments. (**d**) SIM images represent two independent experiments.

The observation that serum triggers rapid OM damage without interfering with the IM suggests that human complement can allow influx of antimicrobial agents that are normally ineffective against Gram-negatives. To study whether there is a window for antibiotics to improve bacterial killing by serum, we studied whether complement may sensitize *E. coli* for the lantibiotic nisin. Nisin kills a range of Gram-positive bacteria by binding to lipid II on the cytoplasmic membrane to disturb PG synthesis and by forming pores in the IM^20–23^. Since it is a large (3.4 kDa) molecule, nisin cannot pass the outer membrane of Gram-negative bacteria^24–26^. Indeed, we confirmed that nisin could not induce Sytox influx in *E. coli* (Fig. 2a). However, nisin efficiently permeabilized the *E. coli* inner membrane in the presence of sublethal concentrations of human serum (0.3%). In addition, whereas 1% serum was sufficient to eventually destabilize the inner membrane of *E. coli*, inner membrane damage was much faster and more efficient when serum and nisin were combined (Fig. 2a). Flow cytometry analysis confirmed that 0.3% serum sensitized the entire bacterial population to nisin concentrations of 1 µg/ml and higher (Fig. 2b,c). Microscopic imaging of bacteria treated with serum or serum and nisin confirmed these observations, showing no effect of nisin on mCherry leakage, but an increase in Sytox positive bacteria when serum and nisin were present together (Fig. 2d).

To study whether the MAC indeed allows nisin to pass the OM, we determined how nisin affects OM and IM damage simultaneously in time. As expected, nisin itself showed no additional effect on OM permeabilization in the presence of serum when compared to bacteria treated with serum only (Fig. 2e). However, nisin rapidly induced IM permeabilization when the OM was damaged by serum (Fig. 2e). In another setup to show that the activity of nisin requires OM permeabilization by the MAC, we blocked MAC formation at different time points using the C5 inhibitor OmCI. Using this approach, we generated bacteria with a damaged OM, but with an intact IM. Analysis of cells after 30 minutes of incubation showed that mCherry leakage is not influenced by nisin (Suppl. Fig. 2). In contrast, nisin damages the IM in mCherry low bacteria that have a perturbed OM (Suppl. Fig. 2). The disruption of the IM correlated with reduced bacterial viability, whereas OM damage itself was not sufficient for bacterial killing (Suppl. Fig. 2). In conclusion, in the presence of serum, nisin can access the periplasm via pores in the OM. In this way, nisin can reach its target sites and rapidly permeabilize the IM of Gram-negative bacteria.

### The MAC allows nisin to kill *E. coli* in serum and whole blood

**Figure 3:**
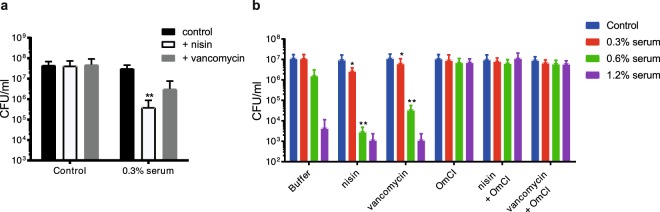
Complement sensitizes *E. coli* for killing by nisin and vancomycin. (**a**) Bacterial viability of *E. coli* MG1655 (OD_600nm_ = 0.01) treated with 0 and 0.3% serum in the presence or absence of 10 µg/ml nisin or vancomycin for 60 min at 37 °C. Samples were serially diluted and plated to analyze the number of surviving bacteria. (**b**) Bacterial viability of *E. coli* MG1655, treated with 0-0.3-0.6-1.2% freshly isolated blood from healthy volunteers in combination with buffer or 10 µg/ml nisin or vancomycin for 60 minutes at 37 °C. Complement specificity was confirmed by adding 10 µg/ml of the MAC inhibitor OmCI. Data represent mean ± SD of three individual experiments. A ratio paired Student’s t-test was used to compare samples in which serum and antibiotics were combined to the ones treated with the same concentration of serum alone. Significant differences are indicated with asterisks (*p < 0.05, **p < 0.01).

To test whether the increase in inner membrane damage upon combination of serum and nisin also leads to more efficient bacterial killing, we evaluated the impact on bacterial viability. In addition, we included the clinically used antibiotic vancomycin, which interferes with peptidoglycan synthesis, to test whether serum can synergize with a broader range of antibiotics. Incubating bacteria with 0.3% serum in the presence of 10 µg/ml nisin or vancomycin resulted in increased killing on plate (Fig. 3a). In a whole blood assay we observed similar effects, both nisin and vancomycin enhanced killing of *E. coli* in the presence of freshly isolated blood (Fig. 3b). Using the complement inhibitor OmCI, we verified that this effect was MAC-mediated. Overall, we conclude that complement can sensitize bacteria to nisin, which can then efficiently trigger IM damage and bacterial killing of the laboratory *E. coli* strain MG1655 and potentially more Gram-negative bacteria.

### Complement sensitizes various Gram-negative clinical isolates to nisin and vancomycin

**Figure 4:**
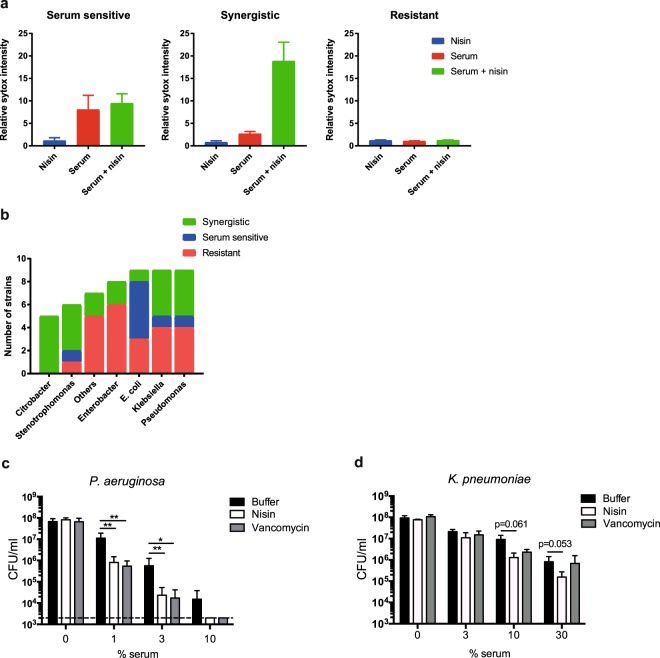
Complement sensitizes various Gram-negative clinical isolates to nisin and vancomycin. (**a**) Inner membrane damage (relative Sytox intensity) of a variety of Gram-negative clinical isolates, treated with 3% serum, 10 µg/ml nisin or both in the presence of Sytox green. Fluorescence was analyzed after 30 min at 37 °C using a microplate fluorometer. The fold increase of the Sytox signal compared to the background was calculated, upon which the isolates were classified into different groups. Left panel: example of a serum sensitive *P. aeruginosa* strain in which inner membrane permeabilization occurs within 30 minutes when treated with serum only. Middle panel: example of synergy between serum and nisin in *S. maltophilia*. Right panel: example of an *E. cloacae* strain that belongs to the resistant group, no IM permeabilization with serum, nisin or the combination of both. (**b**) 53 Gram-negative isolates were analyzed as described above and classified as being serum sensitive, synergistic or resistant. Serum sensitive strains show an average increase of at least 2-fold in Sytox signal over background. In the synergy group, strains have an average increase of at least 1.5-fold when treated with serum and nisin compared to serum only. When no increase in Sytox signal was measured after 30 minutes, isolates were classified as resistant. (**c**,**d**) Viability of a *P. aeruginosa* and *K. pneumoniae* strain incubated with different serum concentrations in the presence or absence of 10 µg/ml nisin or vancomycin for 1 hour at 37 °C. (**a**,**c** and **d**) Data represent mean ± SD of three independent experiments. (**c**,**d**) Data were analyzed by a Student’s t-test and significant differences are indicated with asterisks (*p < 0.05, **p < 0.01).

To translate our findings to clinically relevant bacteria, we studied whether serum can also sensitize clinical isolates to nisin. We screened a collection of 53 Gram-negative patient isolates (including *C. freundii*, *S. maltophilia*, *Enterobacter* spp., *E. coli*, *Klebsiella* spp., *P. aeruginosa* and others (Suppl. Table 1)) that were isolated from different body sites. Clinical isolates were exposed to 3% serum, 10 µg/ml nisin or a combination of both for 30 minutes, after which inner membrane damage was analyzed in our multi-well assay. The tested strains were divided into three categories dependent on their sensitivity to each condition: serum sensitive, synergistic or resistant strains. An example of each category is shown in Fig. 4a. We identified 16 strains (28% of total) that were susceptible to serum alone (Fig. 4b and Suppl. Table 1). Nisin had no additional effect on 8 of these 16 strains (15% of total, classified as serum sensitive), whereas nisin increased IM damage in 8 of these strains (classified as synergistic). Interestingly, 14 strains (26% of total) were only susceptible to the combination of serum and nisin (classified as synergistic). 42% of the tested strains (mostly *Enterobacter* isolates) were completely resistant to serum, nisin or a combination thereof (Fig. 4b). We speculate that these bacteria are relatively resistant to complement-mediated outer membrane damage. Taken together, we found a synergistic effect between complement and nisin in 42% of the tested clinical isolates (all *C. freundii*, 66% of the *S. maltophilia* isolates and 44% of the tested *P. aeruginosa* and *Klebsiella* isolates). These data show that complement can sensitize a variety of Gram-negative species for nisin, which is normally inactive against these strains.

Two isolates (*P. aeruginosa* and *K. pneumoniae*) in which the combination of serum and nisin enhanced IM damage were analyzed in more detail for synergy between serum and nisin or vancomycin. In the presence of serum, nisin induced inner membrane damage in the tested strains in a dose-dependent manner (Suppl. Fig. 3a–d). As for nisin, vancomycin alone showed no activity towards these Gram-negative bacteria (Fig. 4c,d). In concordance with the increase in IM damage, nisin and vancomycin more efficiently killed the *P. aeruginosa* clinical isolate in the presence of serum (Fig. 4c). For the *K. pneumoniae* isolate, nisin and vancomycin showed a less pronounced additive effect in bacterial killing compared to serum only (Fig. 4d). In summary, complement sensitizes Gram-negative clinical isolates to killing by nisin and vancomycin, which are typically not active against such strains.

### Activity of nisin in multi-drug resistant clinical blood isolates

**Figure 5:**
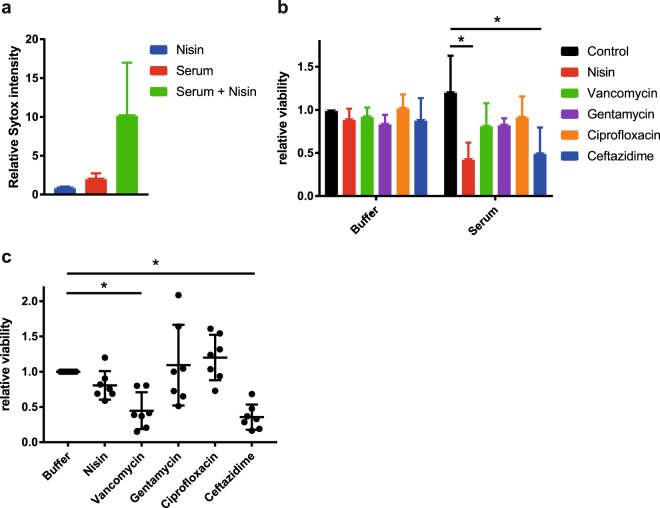
Complement sensitizes multi-drug resistant clinical blood isolates to antibiotics. (**a**) Inner membrane damage (relative Sytox intensity) of *K. pneumoniae* strain 24A013 after 2 hours of exposure to 10 µg/ml nisin, 30% serum or a combination of both. (**b**) Synergy between serum and antibiotics: viability of *K. pneumoniae* strain 24A013 exposed to buffer or 10 µg/ml of different antibiotics in the presence or absence of 60% serum for 3 hours at 37 °C. Samples were serially diluted and viability was determined by counting the colony forming units. CFU counts were normalized to buffer controls. (**c**) Synergy between antibiotics and blood: relative viability of *K. pneumoniae* strain 24A013 exposed to buffer or 10 µg/ml antibiotics in the presence or absence of 60% blood. Samples in which blood and antibiotics were combined were normalized per donor for the ones treated with 60% blood only. (**a**,**b**) Data represent mean ± SD of three independent experiments. (**c**) Data points are shown for seven individual donors. (**b**,**c**) Data were analyzed by a one-way ANOVA and significant differences are indicated with asterisks (*p < 0.05).

Finally, we selected nine multi-drug resistant (MDR) Gram-negative isolates to test whether complement can also sensitize these strains to Gram-positive specific antibiotics. The selected isolates were all resistant to at least one or more compound of three different classes of antibiotics (cephalosporin, aminoglycosides and fluoroquinolone). When incubated with 3% serum, all isolates maintained their IM integrity for at least half an hour (Suppl. Fig. 4a). Three of these strains were sensitized to nisin in the presence of 3% serum as evident by an increase in IM damage (Suppl. Fig. 4a). To test whether the other MDR blood isolates required higher serum concentrations or longer incubation times to induce pore formation, we repeated the screen with 30% serum for 2 hours. Indeed, 30% serum more efficiently induced IM damage in almost all of the MDR isolates (Suppl. Fig. 4b). In three of these isolates, the combination of serum and nisin caused an increase in IM damage compared to serum alone. We selected a *K. pneumoniae* strain that was fully resistant to 30% serum, but sensitive to the combination of serum and nisin (Fig. 5a) to further study bacterial killing by serum in combination with a panel of antibiotics. As expected, the selected isolate was resistant to all the individually tested antibiotics (Fig. 5b). Resistance to vancomycin, gentamycin (an aminoglycoside) and ciprofloxacin (a fluoroquinolone) was not affected by the presence of serum (Fig. 5b). In contrast, the combination of serum and nisin as well as serum and ceftazidime decreased the number of surviving bacteria (Fig. 5b).

To more closely mimic a bloodstream infection, we performed the same assay in freshly isolated blood from 7 different healthy donors. As for serum, the tested strain maintained its resistance to killing by gentamycin and ciprofloxacin in the presence of blood (Fig. 5c). However, combining whole blood with vancomycin and ceftazidime showed a significant drop in viable bacteria, which was present in all 7 donors (Fig. 5c). In contrast to serum, whole blood did not significantly sensitize the tested *K. pneumoniae* strain to killing by nisin, although in 6 out of 7 donors a decrease in bacterial survival was visible. In conclusion, complement can enhance the killing of multi-drug resistant bloodstream isolates by different antibiotics.

## Discussion/Conclusion

The development of novel antibiotics against Gram-negative bacteria is severely hampered by the outer membrane barrier. Whereas 12 out of 15 newly developed antibiotics in the past years were active against Gram-positives, only four had *in vitro* activity against Gram-negative bacteria^5^. The results presented in this study indicate that human complement sensitizes Gram-negative bacteria to antibiotics that are currently considered to be specific for Gram-positives. This action depends on the Membrane Attack Complex (MAC). We recently showed that proper insertion of the MAC into the outer membrane triggers inner membrane damage, which is essential for killing^17^. However, the dynamics of membrane permeabilization by the MAC remained unclear. We here show that the MAC rapidly damages the bacterial outer membrane and only after a delay the bacterial inner membrane. This provides a window for antibiotics to cross the outer membrane barrier and kill Gram-negative bacteria. Although it remains to be elucidated how the MAC eventually damages the bacterial inner membrane, we speculate that this is triggered by extensive outer membrane damage.

The idea that outer membrane permeabilization allows access of larger antimicrobial compounds to underlying structures is in line with previous studies showing that chemical outer membrane perturbing agents sensitize Gram-negative bacteria to several antibiotics^25,27–31^. Furthermore, analogues of naturally impermeable antibiotics with chemical alterations that enhance porin-mediated transport over the outer membrane (e.g. increased lipophilicity, charge or the addition of side groups) show increased activity against Gram-negatives^32–34^. Importantly, since complement proteins are present in most body fluids, our study suggests that several classes of antibiotics that appear ineffective against Gram-negative bacteria in standard *in vitro* assays may in fact be functional *in vivo*, without the need of additional chemical OM destabilizers. Antibiotic activity is normally analyzed in the presence of artificial nutrient rich media that does not mimic a natural environment during infection, and may thus underestimate the *in vivo* effect of the tested compound^35^. Evaluating these and existing antibiotics in the presence of human complement may uncover antimicrobial activity towards certain Gram-negative bacteria, which would not be identified using standard testing procedures. Furthermore, combinations may lower the effective antibiotic concentration as shown for a serum sensitive *E. coli* strain, which was killed by a lower gentamycin concentration in the presence of serum^36^. The here-described multi-well inner membrane permeabilization assay provides an easy platform to screen for potential synergy between serum and antibiotics on clinical isolates. Since nisin efficiently forms pores in the inner membrane when the outer membrane is disrupted, this could function as an initial screening tool to measure whether patient serum may sensitize clinical isolates to antibiotics. The observed synergy of serum with vancomycin indicates that a broad range of antibiotics can be potentiated by complement.

The ability of complement to potentiate antibiotics for Gram-negative bacteria suggests that infections with these bacteria could be treated with Gram-positive-specific antibiotics. However, since several pathogenic bacteria have evolved mechanisms to resist complement lysis^37,38^, we questioned whether these antibiotics would also work on clinical isolates. Screening of a broad range of clinical isolates revealed that, in our experimental conditions, 70% of the patient-derived Gram-negative bacteria were insensitive for lysis by human serum alone. To our surprise, the IM of 42% of the isolates was more efficiently damaged by a combination of nisin and serum. Of these isolates, 26% was insensitive to IM damage by serum alone, indicating that human complement may not directly kill these bacteria but still form pores in the OM that grant access for naturally impermeable antibiotics. For those strains resistant to the combination of serum and nisin, a higher serum concentration may be required to damage the outer membrane. Alternatively, resistant strains in which the MAC fails to properly insert into the outer membrane may benefit from outer membrane permeabilizing agents, which could both enhance MAC insertion into the outer membrane and allow antibiotics to more efficiently cross the outer membrane^31,39^. So whereas complement alone can damage both the outer and inner membrane of *E. coli* MG1655 and some clinical isolates, a subset of the strains is only sensitive to complement-dependent outer membrane, but not to inner membrane destabilization in our experimental setup.

In addition, we found that serum can sensitize MDR strains to nisin and ceftazidime, an antibiotic that also interferes with cell wall synthesis. Although the effect of nisin in whole blood was less pronounced, we observed a (non-significant) drop in viable bacteria in 6 out of 7 tested donors. It is unlikely that nisin is less active in blood due to degrading enzymes, since these are probably also present in serum. Therefore, we are yet unable to explain the difference between nisin functionality in serum and whole blood. In contrast, while serum did not increase bacterial killing of the tested MDR strain by vancomycin, this was the case when vancomycin was combined with whole blood. Also ceftazidime was efficiently potentiated for killing of *K. pneumoniae* in the presence of blood. In contrast to nisin and vancomycin, Gram-negative bacteria are normally slightly susceptible to ceftazidime. However, ceftazidime has a low rate of entry due to its low sensitivity for outer membrane porins^11^. In line with our results, killing of a MDR *P. aeruginosa* is enhanced when ceftazidime is combined with OM disrupting agents such as colistin^40^ or rabbit serum supplemented with arachidonic acid^41^. We here show that human complement, and potentially other blood components, can also increase the killing efficiency of ceftazidime on a MDR *K. pneumoniae*. Altogether, our data implies that complement can especially enhance the efficiency of antibiotics that have low permeation through the outer membrane and are therefore ineffective. In contrast to ceftazidime, fluoroquinolones and aminoglycosides can cross the outer membrane via porins^42,43^. The fact that complement did not enhance bactericidal activity of these antibiotics in our assays confirms that resistance is not dependent on the outer membrane but that other resistance mechanisms are involved. Taken together, the enhancing effect of complement may especially be relevant for antibiotics that lack bactericidal activity due to low permeation through the outer membrane, like erythromycin, clindamycin or rifampicin^31^. This, together with the observed differences between isolates suggest that a ‘personalized’ functional screening of antibiotics on the patient’s isolate in combination with the patient’s serum or blood would be ideal to determine the therapeutic regimen. In the future, when therapeutic antibodies against Gram-negatives are clinically available^44^, a combination therapy of antibodies (driving complement-mediated lysis) and antibiotics may present an effective and tailored option for treating Gram-negative infections.

In conclusion, we show that human complement can effectively sensitize Gram-negative bacteria for antimicrobial agents that are naturally ineffective against these bacteria. Testing antibiotics in the presence of complement may therefore reveal antimicrobial activity towards certain Gram-negative bacteria, which would not be identified using conventional testing procedures and expand the applicability of existing and novel antibiotics.

## Materials and Methods

### Bacterial strain and plasmid preparation

The plasmid pFCcGi containing a constitutive expressed mCherry and an L-arabinose inducible GFP (kindly provided by Sophie Helaine^45^ was modified to direct mCherry to the periplasmic space by adding a pelB leader in front of the mCherry sequence. The new plasmid, pPerimCh, was transformed into MG1655.

### Serum preparation and reagents

For normal human serum preparation, blood was drawn from healthy volunteers and allowed to clot for 15 minutes at RT. After centrifugation (10 min, 4000 rpm), serum was collected, pooled and stored at −80 °C. Heat inactivated (HI) serum was obtained by incubating serum for 30 min at 56 °C. Sera deficient for complement components C8 and C9 were obtained from Complement Technology. OmCI was produced in HEK 293E cells (U-Protein Express, Utrecht) and purified as previously described^19^.

### Bacterial IM destabilization in multi-well Sytox assay

Bacteria were grown to midlog phase (OD_600nm_ ~ 0.5) in Lysogeny Broth (LB) medium, pelleted and resuspended to OD_600nm_ of 1.0 in RPMI 1640 (ThermoFisher) supplemented with 0.05% HSA. Ten-fold diluted bacteria were incubated (at an end concentration of OD_600nm_ ~ 0.05) with 0–10% serum, 10% heat-inactivated serum or 10% serum + 20 µg/ml OmCI. Incubations were done in the presence of 1 µM Sytox Green Nucleic Acid stain (Thermofisher). Fluorescence was measured in a microplate reader (CLARIOstar, Labtech) at 37 °C under non-shaking conditions.

Synergy experiments were performed by incubating bacteria with the indicated concentrations of nisin **(**Handary SA, Brussels) in the presence of serum. The clinical isolates were classified as serum sensitive if the fluorescent signal was at least 2 times higher when treated with 3% serum as compared to the buffer control after 30 minutes of incubation at 37 °C. Strains with a 50% increase in Sytox Green fluorescence when treated with both 3% serum and 10 µg/ml nisin compared to serum only were classified as synergistic. The resistant group contains strains that are insensitive to serum, nisin or a combination thereof. Multi-drug resistant strains were incubated with 3% serum for half an hour or with 30% serum for 2 hours after which similar criteria were applied to classify these strains as being serum sensitive, synergistic or resistant.

### Flow cytometry

Bacteria were grown to midlog phase (OD_600nm_ ~ 0.5) in Lysogeny Broth (LB) medium, pelleted and dissolved to a density of OD_600nm_ = 0.1 in RPMI-0.05% HSA. Bacteria (end concentration OD_600nm_ = 0.05) were incubated with indicated serum concentration in the presence of 5 µM Sytox Blue Nucleic Acid stain (Thermofisher). Flow cytometry analysis was performed using the MACSQuant (Miltenyi biotech) by analyzing 10,000 events per condition, except for measurements in time. Bacteria were gated based on forward and side scatters or in the case of L-arabinose induced MG1655-pPerimCh on GFP. The percentage of Sytox positive cells was determined by analyzing the number of cells that show an increase in Sytox signal based on the negative peak of the buffer control. For MG1655 expressing periplasmic mCherry, the geometric mean of the bacterial population was determined. The data were analyzed in FlowJo.

### Bacterial viability assay

Bacteria were prepared as described above and incubated with buffer, serum or blood (drawn from healthy volunteers) in the presence or absence of antibiotics (10 µg/ml nisin, vancomycin, gentamycin, ciprofloxacin or ceftazidime). For CFU/ml determination, serial dilutions were made in PBS and bacteria were plated onto agar plates followed by colony enumeration after overnight incubation. Relative viability was calculated as the number of CFU/ml relative to the number of CFU/ml at t = 0 or to the buffer control (as indicated in the figure legends).

### Structured Illumination Microscopy

8-well microslide (Ibidi) chambers were washed 3 times with a 500 µl 1 M HCL/70% EtOH solution and rinsed with 500 µl MilliQ (MQ) for 3 times. Chambers were incubated with 150 µl 1 M sodium acetate/0.01 M NaOH and 4 µl Cell Tak solution (Corning) for 20 min (RT). Chambers were rinsed 3 times with MQ and dried. MG1655 with periplasmic mCherry and L-arabinose inducible cytosolic GFP were grown to a midlog phase (OD_600nm_ ~ 0.5) in the presence of 0.1% L-arabinose. Bacteria were washed 3 times in MQ and 150 µl of 4 times concentrated bacteria was added to the chambers. After 30 min, chambers were rinsed 3 times in MQ (avoid drying out) and RPMI/0.05% HSA containing 5 µM Sytox Blue Nucleic Acid Stain (Thermofisher) and 0.5 µM Trolox (Sigma-Aldrich) was added. A T = 0 image was taken, 3% serum or 3% serum and 3 µg/ml nisin was added and bacteria were imaged after 15 and 30 minutes. Images were obtained using the GE Healthcare LifeSciences “Deltavision OMXV4 blaze” microscope using 60x Olympus lens (U-PLAN APO, NA 1.42) and immersion oil 1.516 (Cargille labs). Sytox blue, GFP and mCherry signals were measured using the 405 nm, 488 nm and 561 nm lasers respectively with suited dichroics and emission filter setting of 436/31, 528/48 and 609/37. Reconstructions and registrations were performed using softWoRx (GE-healthcare). Linear adjustments in brightness levels of the images were performed similarly on each sample using FIJI software.

### Statistical testing

Statistical analysis was performed using the ratio paired two-tailed Student’s t-test or a one-way ANOVA. The tests and n-values used to calculate p-values are indicated in the figure legends.

### Ethics statement

Human blood was isolated after informed consent was obtained from all subjects in accordance with the Declaration of Helsinki. Approval was obtained from the medical ethics committee of the UMC Utrecht, The Netherlands.

## Supplementary information

Supplementary information

## Data Availability

All data generated or analyzed during this study is included in the published article and its Supplementary Information.
