# Publisher Correction: Complement-dependent outer membrane perturbation sensitizes Gram-negative bacteria to Gram-positive specific antibiotics

**DOI:** 10.1038/s41598-019-43208-4

**Published:** 2019-05-21

**Authors:** D. A. C. Heesterbeek, N. I. Martin, A. Velthuizen, M. Duijst, M. Ruyken, R. Wubbolts, S. H. M. Rooijakkers, B. W. Bardoel

**Affiliations:** 1 Medical Microbiology, University Medical Center Utrecht, Utrecht University, Utrecht, Netherlands; 2 Institute of Biology Leiden, Leiden University, Leiden, Netherlands; 3 Department of Biochemistry and Cell Biology, Utrecht University, Utrecht, Netherlands

Correction to: *Scientific Reports* 10.1038/s41598-019-38577-9, published online 28 February 2019

In the original version of this Article, a reference was omitted. This reference has now been added as Reference 17 and the original References 17–44 are now listed as References 18–45, respectively.

As a result, in the Introduction section,

“Recently we observed that when the MAC properly forms pores in the outer membrane of *E. coli*, this triggers inner membrane damage and killing (*Heesterbeek et al., accepted for publication in EMBO journal, see attached manuscript)*.”

now reads:

“Recently we observed that when the MAC properly forms pores in the outer membrane of *E. coli*, this triggers inner membrane damage and killing^17^.

In the Methods section, under subheading ‘Complement-mediated outer membrane damage is more efficient than inner membrane damage’,

“In order to study how the MAC kills bacteria, we used a flow cytometry based assay to measure serum-induced outer and inner membrane damage in *E. coli* (*Heesterbeek et al., accepted for publication in EMBO journal, see attached manuscript*).”

now reads:

“In order to study how the MAC kills bacteria, we used a flow cytometry based assay to measure serum-induced outer and inner membrane damage in *E. coli*^17^.”

Finally, in the Discussion/Conclusion section,

“We recently showed that proper insertion of the MAC into the outer membrane triggers inner membrane damage, which is essential for killing (*Heesterbeek et al., accepted for publication in EMBO journal, see attached manuscript)*.”

now reads:

“We recently showed that proper insertion of the MAC into the outer membrane triggers inner membrane damage, which is essential for killing^17^.

This error has been corrected in the PDF and HTML versions of the article.

