# Supplementary material for: Complement-dependent outer membrane perturbation sensitizes Gram-negative bacteria to Gram-positive specific antibiotics
Source: Sci Rep. 2019 Feb 28;9:3074. doi: 10.1038/s41598-019-38577-9 (PMC6395757; doi:10.1038/s41598-019-38577-9)
Supplement: Supplementary file 1 — Supplementary information [file 41598_2019_38577_MOESM1_ESM.pdf]

Supplementary information

Complement-dependent outer membrane perturbation sensitizes Gram-negative bacteria to Gram-positive specific antibiotics

Heesterbeek DAC[1], Martin NI[2], Velthuizen A[1], Duijst M[1], Ruyken M[1], Wubbolts R[3], Rooijakkers SHM[1] and Bardoel BW[1*].

[1]*Medical Microbiology, University Medical Center Utrecht, Utrecht University, Utrecht, Netherlands*

[2] *Department of Chemical Biology and Drug Discovery, Utrecht University, Netherlands*

[3] *Department of Biochemistry and Cell Biology, Utrecht University, Utrecht, Netherlands*

Corresponding author:

*Bart Bardoel: b.w.bardoel-2@umcutrecht.nl

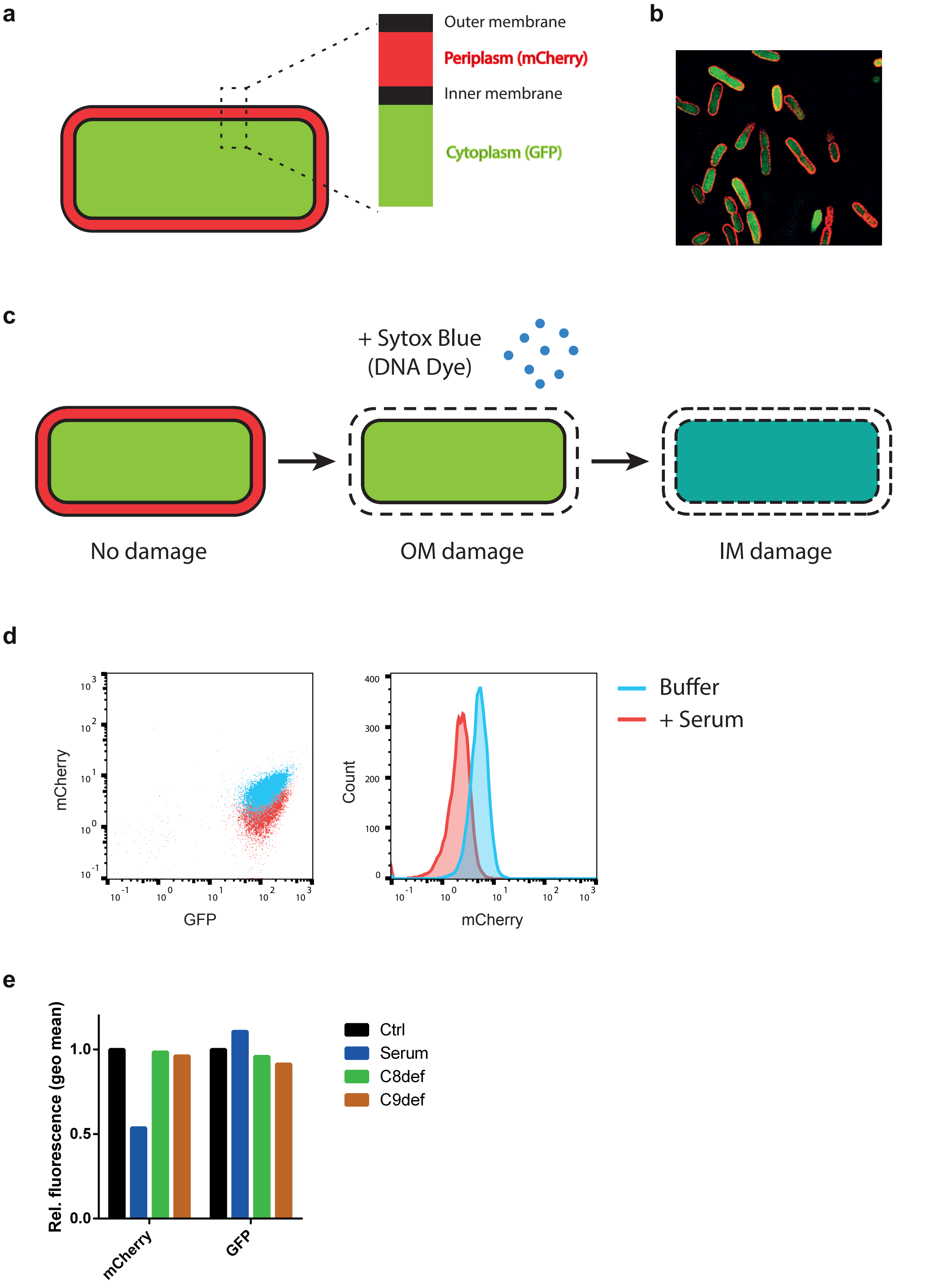

Supplementary figure 1. Analysis of membrane damage using bacteria expressing mCherry in the periplasm and GFP in the cytoplasm. A) Schematic overview of MG1655-pPerimCh bacteria with cytoplasmic GFP (green) and periplasmic mCherry (red). Cell membranes are depicted in black. B) Structured illumination microscopy image to verify localization of cytoplasmic GFP (green) and periplasmic mCherry (red) in *E. coli*. C) Schematic overview of a membrane permeabilization assay setup in which outer membrane damage is measured via leakage of mCherry from the periplasm, and where inner membrane damage is measured via Sytox influx and GFP leakage. D) MG1655-pPerimCh was exposed to 3% serum for 30 minutes at RT, after which mCherry and GFP intensities were measured by flow cytometry. Graphs show data from a representative experiment of at least three independent experiments. E) MG1655-pPerimCh was cultured in the presence of 0.1% L-arabinose to induce cytosolic GFP. Bacteria were treated with buffer, 1% serum, 1% C8 depleted serum or 1% C9 depleted serum for 30 minutes at 37°C. Cells were analyzed for GFP and mCherry signal by flow cytometry.

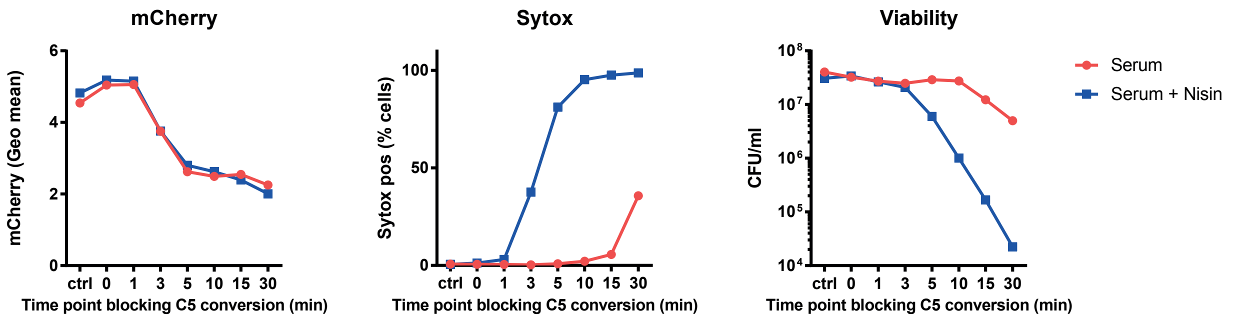

Supplementary figure 2. MAC-dependent outer membrane damage sensitizes *E. coli* to nisin. MG1655-pPerimCh was incubated with 3% serum in presence or absence of 3 µg/ml nisin. After 0-1-3-5-10-15-30 minutes 10 µg/ml OmCI was added to the incubation mixture to block MAC formation. After 30 minutes bacteria were analyzed for mCherry intensity and percentage Sytox blue positive cells by flow cytometry. The same samples were diluted and analyzed for viability by determining the number of colony forming units. A representative graph of at least three independent experiments is shown.

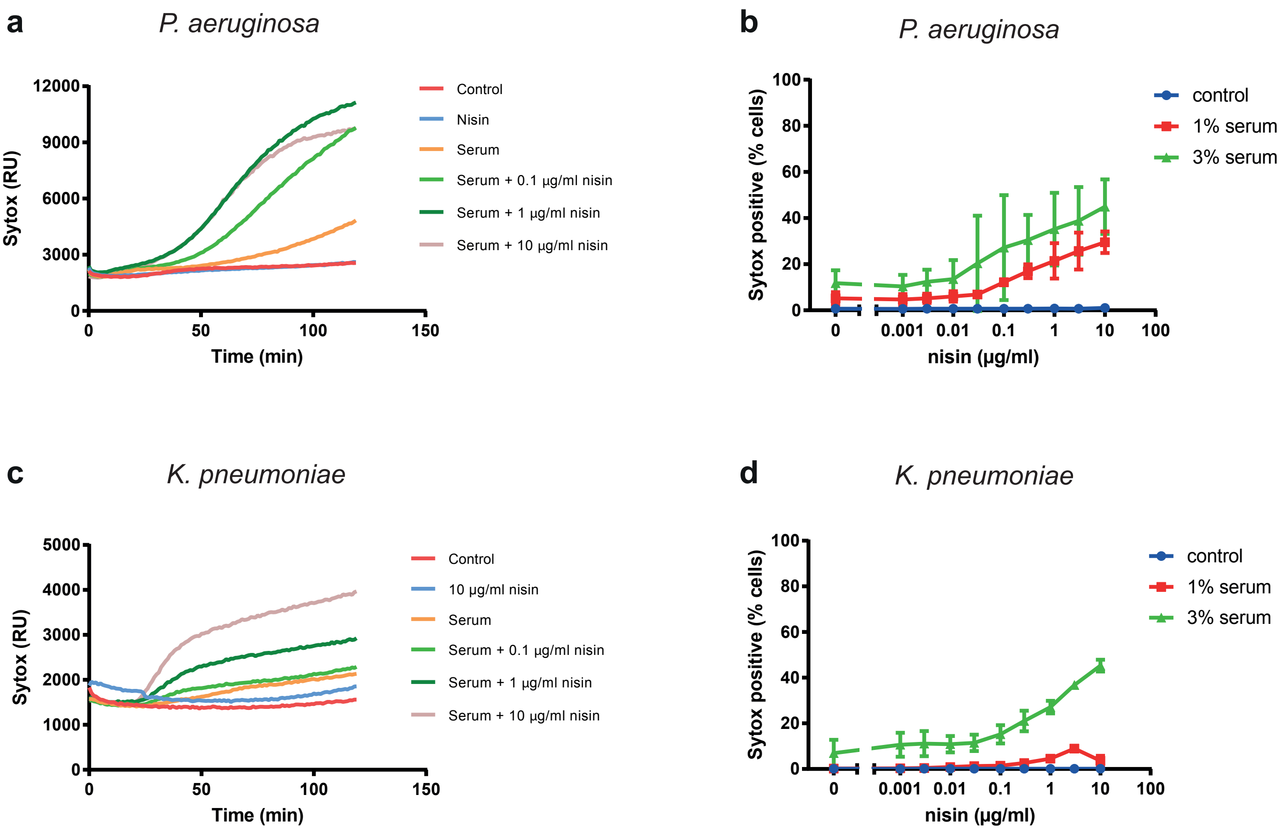

Supplementary figure 3. Complement sensitizes Gram-negative bacteria for inner membrane damage by nisin. A) Inner membrane damage (Sytox intensity) of *P. aeruginosa* 547966.3 and C) *K. pneumoniae* 5709879.1 treated with buffer or serum (1% or 3% respectively) in the presence or absence of a concentration range of nisin. Inner membrane damage was analyzed by measuring Sytox intensity in a microplate fluorometer. Graphs show data from a representative experiment of at least three independent experiments. B) Inner membrane damage (% Sytox positive) of *P. aeruginosa* 547966.3 and D) *K. pneumoniae* 5709879.1 treated with buffer, 1% or 3% serum in the presence of a concentration range of nisin. Sytox intensity was measured by flow cytometry. Data represent mean ± SD of three independent experiments.

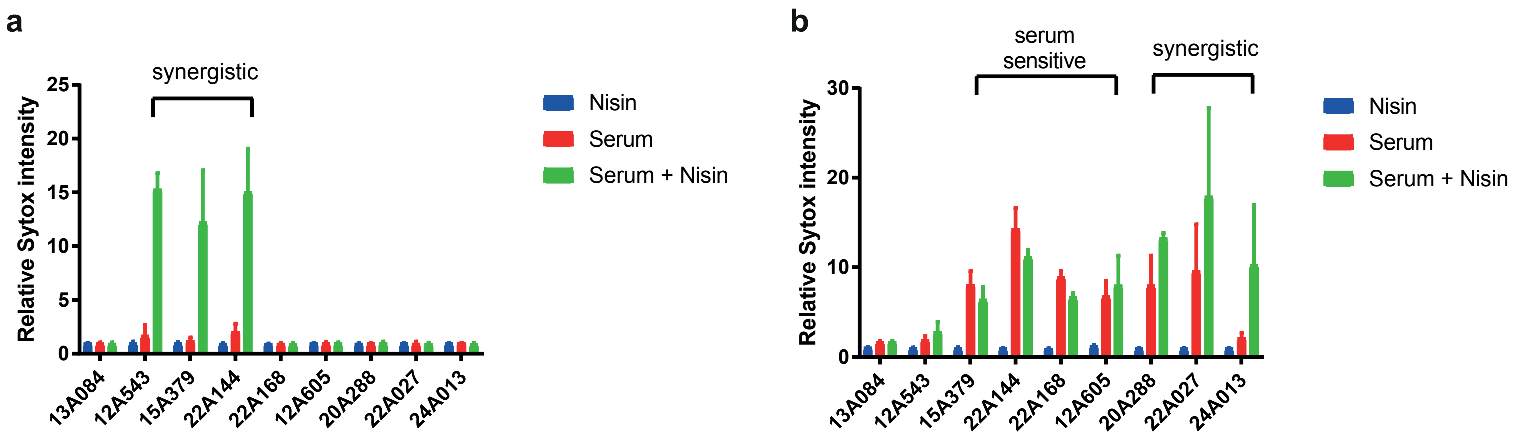

Supplementary figure 4. Complement sensitizes multi-drug resistant clinical isolates to inner membrane damage by nisin. A) Relative Sytox intensity of a panel of 9 MDR strains after 30 minutes of exposure to 10 µg/ml nisin, 3% serum or a combination of both. B) Relative Sytox intensity of a panel of 9 MDR strains after 2 hours of exposure to 10 µg/ml nisin, 30% serum or a combination of both. Data represent mean ± SD of three independent experiments.

Supplementary table 1. Sensitivity of Gram-negative clinical isolates to nisin in the absence and presence of serum. All Sytox fluorescence values for the clinical isolates that were used to determine whether strains are sensitive to nisin, serum or the combination of serum and nisin. The summary of these values is depicted in Fig. 4B.

	N=1	Control	Nisin	Serum	Serum + Nisin
Citrobacter freundii 547126.2 sputum	1	521	1417	1759	7025
Citrobacter freundii 548359.2 Sputum	2	776	326	794	4647
Citrobacter freundii 552036.2 katheter urine	3	1552	1734	2304	3189
Citrobacter freundii 551964.1 urine midstream	4	1392	1835	1754	2210
Citrobacter freundii 552955.1 urine	5	570	514	5845	16725
Enterobacter aerogenes 548189.1 throat	6	310	857	1336	6315
Entrobacter cloacae 547651.1 urine midstream	7	986	997	839	4538
Enterobacter cloacae 549052.2 sputum	8	771	451	734	575
Enterobacter cloacae 552270.1 faeces	9	1530	1578	1510	1666
Enterobacter cloacae 552275.2 faeces	10	1425	1659	1567	1755
Enterobacter cloacae 566566.1 exudate	11	767	798	1113	1225
Enterobacter cloacae 567393.2 urine	12	639	644	996	935
Enterobacter cloacae 567515.2	13	894	638	1027	1007
E.coli 547655.1 katheter urine	14	276	1182	977	670
E.coli 547654.1 katheter urine	15	424	798	1146	572
E.coli 547563.1 urine midstream	16	800	827	9574	8790
E. coli 552059.1 urine midstream	17	1390	1729	1672	1542
E. coli 552060.1 urine midstream	18	1722	1736	1674	1734
E. coli 552866.1 urine	19	976	1862	563	1894
E. coli 552912.1 urine	20	1017	1016	1993	1932
E. coli 567705.1 rectum	21	320	975	1412	1387
E. coli 566989.1 sputum	22	1408	342	10508	11977
Klebsiella oxytoca 548359.3 sputum	23	983	812	2596	9047
Klebsiella oxytoca 567267.1 throat	24	652	622	645	2668
Klebsiella oxytoca 566829.2 ascites	25	917	808	1259	1594
Klebsiella oxytoca 566989.2 sputum	26	850	730	1146	912
Klebsiella oxytoca 566951.2 sputum	27	382	432	1113	1235
Klebsiella pneunomiae 567709.1 throat	28	804	841	3363	13399
Klebsiella pneunomiae 567570.1 rectum	29	266	673	1079	1289
Klebsiella pneunomiae 567702.1 rectum	30	268	777	1814	11121
Klebsiella oxytoca 571288.2	31	799	337	1112	1448
Proteus mirabilis 552764.1 urine midstream	32	1355	1560	1494	1703
Proteus mirabilis 566784.1 drainvocht	33	722	444	965	996
Serratia marcescens 567055.1 throat	34	685	683	915	919
Pseudomonas aeruginosa 547966.3 Sinus	35	2158	1567	3752	19592
Pseudomonas aeruginosa 566061.1 rectum	36	630	544	1145	1297
Pseudomonas aeruginosa 566674.2 urine	37	835	810	1865	1037
Pseudomonas aeruginosa 567414.1 cathetertip **	38	2435	1262	10971	26507
Pseudomonas aeruginosa 567023.1 throat	39	475	786	474	1102
Pseudomonas aeruginosa 566829.3 ascites	40	829	207	1118	1111
Pseudomonas aeruginosa 566671.3 urine	41	867	451	1573	11527
Pseudomonas aeruginosa 567023.2	42	1130	594	1912	7386
Pseudomonas aeruginosa 567003.1	43	935	570	1373	4817
Stenotrophomonas maltophilia 547126.1 sputum	44	1933	796	1641	18166
Stenotrophomonas maltophilia 567270.1 exudate	45	799	933	1856	14523
Stenotrophomonas maltophilia 566954.1 sputum	46	840	419	2728	12335
Stenotrophomonas maltophilia 567010.1	47	1388	1255	1372	1922
Stenotrophomonas maltophilia 570610.1	48	240	1111	1172	341
Chryseobacterium gleum 547472.4 sputum	49	664	860	1089	410
Acinetobacter radioresistens 566973.1 exudate	50	1186	1403	1552	8780
Hafnia alvei 567060.1 faeces	51	718	385	2634	13500
Morganella morganii 566829.1 ascites	52	796	1009	1062	1247
Stenotrophomonas maltophilia 570287.3**	53	2502	2862	8744	20122

Ratio

Control	Nisin	Serum	Serum + Nisin
1,00	2,72	3,38	13,48
1,00	0,42	1,02	5,99
1,00	1,12	1,48	2,05
1,00	1,32	1,26	1,59
1,00	0,90	10,25	29,34
1,00	2,76	4,31	20,37
1,00	1,01	0,85	4,60
1,00	0,58	0,95	0,75
1,00	1,03	0,99	1,09
1,00	1,16	1,10	1,23
1,00	1,04	1,45	1,60
1,00	1,01	1,56	1,46
1,00	0,71	1,15	1,13
1,00	4,28	3,54	2,43
1,00	1,88	2,70	1,35
1,00	1,03	11,97	10,99
1,00	1,24	1,20	1,11
1,00	1,01	0,97	1,01
1,00	1,91	0,58	1,94
1,00	1,00	1,96	1,90
1,00	3,05	4,41	4,33
1,00	0,24	7,46	8,51
1,00	0,83	2,64	9,20
1,00	0,95	0,99	4,09
1,00	0,88	1,37	1,74
1,00	0,86	1,35	1,07
1,00	1,13	2,91	3,23
1,00	1,05	4,18	16,67
1,00	2,53	4,06	4,85
1,00	2,90	6,77	41,50
1,00	0,42	1,39	1,81
1,00	1,15	1,10	1,26
1,00	0,61	1,34	1,38
1,00	1,00	1,34	1,34
1,00	0,73	1,74	9,08
1,00	0,86	1,82	2,06
1,00	0,97	2,23	1,24
1,00	0,52	4,51	10,89
1,00	1,65	1,00	2,32
1,00	0,25	1,35	1,34
1,00	0,52	1,81	13,30
1,00	0,53	1,69	6,54
1,00	0,61	1,47	5,15
1,00	0,41	0,85	9,40
1,00	1,17	2,32	18,18
1,00	0,50	3,25	14,68
1,00	0,90	0,99	1,38
1,00	4,63	4,88	1,42
1,00	1,30	1,64	0,62
1,00	1,18	1,31	7,40
1,00	0,54	3,67	18,80
1,00	1,27	1,33	1,57
1,00	1,14	3,49	8,04

N=2

Control	Nisin	Serum	Serum + Nisin
3356	2023	1679	7099
1286	1209	1607	7389
3339	2838	2157	11628
2052	2259	1437	6035
1343	1281	4040	33931
1085	1042	1100	4007
1732	1829	2305	24448
1466	1427	1096	1091
1200	1067	998	968
1661	2045	1871	2098
1470	1704	2038	2050
1455	1281	1051	1041
1410	1496	1026	988
931	883	1734	6008
914	871	1897	1614
1119	1064	11288	25907
1307	1155	1515	1056
1300	1105	1002	961
956	895	1167	933
1065	1019	1740	4695
1083	963	930	896
1107	1037	8933	14480
1161	1012	1249	4067
821	760	854	974
1018	979	976	1330
971	931	1166	916
986	904	1276	865
847	774	881	2591
883	813	814	819
855	802	1241	2517
1115	1045	945	1029
746	700	1231	811
806	755	809	829
815	754	796	787
3308	3883	5075	28408
1236	1197	950	950
1629	1401	1086	1038
4509	7576	41092	31903
1472	1483	1391	1538
1238	1325	1238	1292
2257	3232	4239	13219
1478	1664	4258	11945
1129	1221	1631	4126
1321	1103	4046	14060
1214	1270	1837	15710
788	770	1778	14930
1671	1485	1888	1977
914	949	963	1400
734	679	798	788
3533	2341	1850	4680
1094	1110	4392	30444
1074	1034	1602	2021
3193	2777	10951	19934

Ratio

Control	Nisin	Serum	Serum + Nisin
1,00	0,60	0,50	2,12
1,00	0,94	1,25	5,75
1,00	0,85	0,65	3,48
1,00	1,10	0,70	2,94
1,00	0,95	3,01	25,27
1,00	0,96	1,01	3,69
1,00	1,06	1,33	14,12
1,00	0,97	0,75	0,74
1,00	0,89	0,83	0,81
1,00	1,23	1,13	1,26
1,00	1,16	1,39	1,39
1,00	0,88	0,72	0,72
1,00	1,06	0,73	0,70
1,00	0,95	1,86	6,45
1,00	0,95	2,08	1,77
1,00	0,95	10,09	23,15
1,00	0,88	1,16	0,81
1,00	0,85	0,77	0,74
1,00	0,94	1,22	0,98
1,00	0,96	1,63	4,41
1,00	0,89	0,86	0,83
1,00	0,94	8,07	13,08
1,00	0,87	1,08	3,50
1,00	0,93	1,04	1,19
1,00	0,96	0,96	1,31
1,00	0,96	1,20	0,94
1,00	0,92	1,29	0,88
1,00	0,91	1,04	3,06
1,00	0,92	0,92	0,93
1,00	0,94	1,45	2,94
1,00	0,94	0,85	0,92
1,00	0,94	1,65	1,09
1,00	0,94	1,00	1,03
1,00	0,93	0,98	0,97
1,00	1,17	1,53	8,59
1,00	0,97	0,77	0,77
1,00	0,86	0,67	0,64
1,00	1,68	9,11	7,08
1,00	1,01	0,94	1,04
1,00	1,07	1,00	1,04
1,00	1,43	1,88	5,86
1,00	1,13	2,88	8,08
1,00	1,08	1,44	3,65
1,00	0,83	3,06	10,64
1,00	1,05	1,51	12,94
1,00	0,98	2,26	18,95
1,00	0,89	1,13	1,18
1,00	1,04	1,05	1,53
1,00	0,93	1,09	1,07
1,00	0,66	0,52	1,32
1,00	1,01	4,01	27,83
1,00	0,96	1,49	1,88
1,00	0,87	3,43	6,24

N=3

Control	Nisin	Serum	Serum + Nisin
2582	2490	2317	5933
1357	1147	2042	6760
1933	1578	1320	6819
1801	1634	2223	4017
1212	1073	2737	19518
1182	1075	1328	2857
1746	1831	2368	33498
1405	1297	1088	1153
960	883	991	962
1513	2017	1582	1962
1925	2209	1862	2492
1219	1065	1321	1208
1248	1095	1019	1053
1150	893	1102	3362
1036	958	1141	1398
1127	978	13279	26344
1501	1332	1106	1059
1682	1503	1207	1126
948	920	975	965
1170	1048	1373	3956
916	821	1095	892
1395	1180	4891	15432
1122	993	1176	4543
931	826	907	1281
1141	1090	1049	1537
1100	1036	1017	1013
1249	1139	1006	1139
954	909	975	1816
990	918	918	911
908	860	931	2280
1150	1041	983	1013
1040	985	945	1276
752	714	885	894
887	812	884	949
1412	1843	2900	29580
1087	948	933	932
1032	1006	1469	977
2579	3681	27302	27102
1336	1229	1560	1191
904	970	945	1000
1439	1434	1595	4890
1411	1303	2335	8848
1002	993	1151	3182
1414	1356	5514	29397
1112	1034	1178	14513
914	945	2374	21053
1247	1200	1143	1355
1182	1087	1063	1094
798	943	1224	1106
1335	1528	1376	5217
975	918	3000	30383
827	793	943	1791
1562	1458	8579	26019

Ratio

Control	Nisin	Serum	Serum + Nisin
1,00	0,96	0,90	2,30
1,00	0,85	1,50	4,98
1,00	0,82	0,68	3,53
1,00	0,91	1,23	2,23
1,00	0,89	2,26	16,10
1,00	0,91	1,12	2,42
1,00	1,05	1,36	19,19
1,00	0,92	0,77	0,82
1,00	0,92	1,03	1,00
1,00	1,33	1,05	1,30
1,00	1,15	0,97	1,29
1,00	0,87	1,08	0,99
1,00	0,88	0,82	0,84
1,00	0,78	0,96	2,92
1,00	0,92	1,10	1,35
1,00	0,87	11,78	23,38
1,00	0,89	0,74	0,71
1,00	0,89	0,72	0,67
1,00	0,97	1,03	1,02
1,00	0,90	1,17	3,38
1,00	0,90	1,20	0,97
1,00	0,85	3,51	11,06
1,00	0,89	1,05	4,05
1,00	0,89	0,97	1,38
1,00	0,96	0,92	1,35
1,00	0,94	0,92	0,92
1,00	0,91	0,81	0,91
1,00	0,95	1,02	1,90
1,00	0,93	0,93	0,92
1,00	0,95	1,03	2,51
1,00	0,91	0,85	0,88
1,00	0,95	0,91	1,23
1,00	0,95	1,18	1,19
1,00	0,92	1,00	1,07
1,00	1,31	2,05	20,95
1,00	0,87	0,86	0,86
1,00	0,97	1,42	0,95
1,00	1,43	10,59	10,51
1,00	0,92	1,17	0,89
1,00	1,07	1,05	1,11
1,00	1,00	1,11	3,40
1,00	0,92	1,65	6,27
1,00	0,99	1,15	3,18
1,00	0,96	3,90	20,79
1,00	0,93	1,06	13,05
1,00	1,03	2,60	23,03
1,00	0,96	0,92	1,09
1,00	0,92	0,90	0,93
1,00	1,18	1,53	1,39
1,00	1,14	1,03	3,91
1,00	0,94	3,08	31,16
1,00	0,96	1,14	2,17
1,00	0,93	5,49	16,66

Average ratio of three experiments

Control	Nisin	Serum	Serum + Nisin	Serum+ nisin/serum	Serum sensitive	Synergistic
1,0	1,4	1,6	6,0	3,7		X
1,0	0,7	1,3	5,6	4,4		X
1,0	0,9	0,9	3,0	3,2		X
1,0	1,1	1,1	2,3	2,1		X
1,0	0,9	5,2	23,6	4,6	X	X
1,0	1,5	2,1	8,8	4,1	X	X
1,0	1,0	1,2	12,6	10,7		X
1,0	0,8	0,8	0,8	0,9		
1,0	0,9	1,0	1,0	1,0		
1,0	1,2	1,1	1,3	1,2		
1,0	1,1	1,3	1,4	1,1		
1,0	0,9	1,1	1,1	0,9		
1,0	0,9	0,9	0,9	1,0		
1,0	2,0	2,1	3,9	1,9	X	
1,0	1,3	2,0	1,5	0,8	X	
1,0	1,0	11,3	19,2	1,7	X	
1,0	1,0	1,0	0,9	0,8		
1,0	0,9	0,8	0,8	1,0		
1,0	1,3	0,9	1,3	1,4		
1,0	1,0	1,6	3,2	2,0		X
1,0	1,6	2,2	2,0	0,9	X	
1,0	0,7	6,3	10,9	1,7	X	
1,0	0,9	1,6	5,6	3,5		X
1,0	0,9	1,0	2,2	2,2		X
1,0	0,9	1,1	1,5	1,4		
1,0	0,9	1,2	1,0	0,8		
1,0	1,0	1,7	1,7	1,0		
1,0	1,0	2,1	7,2	3,5	X	X
1,0	1,5	2,0	2,2	1,1	X	
1,0	1,6	3,1	15,7	5,1		X
1,0	0,8	1,0	1,2	1,2		
1,0	1,0	1,2	1,2	1,0		
1,0	0,8	1,2	1,2	1,0		
1,0	0,9	1,1	1,1	1,0		
1,0	1,1	1,8	12,9	7,2		X
1,0	0,9	1,1	1,2	1,1		
1,0	0,9	1,4	0,9	0,7		
1,0	1,2	8,1	9,5	1,2	X	
1,0	1,2	1,0	1,4	1,4		
1,0	0,8	1,1	1,2	1,0		
1,0	1,0	1,6	7,5	4,7		X
1,0	0,9	2,1	7,0	3,4	X	X
1,0	0,9	1,4	4,0	2,9		X
1,0	0,7	2,6	13,6	5,2	X	X
1,0	1,0	1,6	14,7	9,0		X
1,0	0,8	2,7	18,9	7,0	X	X
1,0	0,9	1,0	1,2	1,2		
1,0	2,2	2,3	1,3	0,6	X	
1,0	1,1	1,4	1,0	0,7		
1,0	1,0	1,0	4,2	4,4		X
1,0	0,8	3,6	25,9	7,2	X	X
1,0	1,1	1,3	1,9	1,4		
1,0	1,0	4,1	10,3	2,5	X	X

Supplementary table 2. Multi-drug resistant Gram-negative isolates used in this study. The antibiotic resistant profile of the multi-drug resistant Gram-negative isolates. For each strain the minimal inhibitory concentration (in µg/ml) for each antibiotic is shown.

#	Category	Date of isolation	ID number	Centre name	Study objective	Location within hospital	Method species ident	Genus of isolate	Species of isolate	Amikacin	Gentamicin	Tobramycin	Ciprofloxacin	Ofloxacin	Levofloxacin	Sparfloxacin	Gatifloxacin	Trovafloxacin	Grepafloxacin	Nalidixic acid	Tetracycline	Nitrofurantoin	Ampicillin	Trimethoprim/sulfamethoxazole
1	Gram Negative	20NOV1998	12A605	UTRECHT	BLOOD	INTENSIVE CARE	VITEK	KLEBSIELLA	OXYTOCA	2	> 16	16	> 2	> 4	4		4	1	> 2	<=8	> 8	<=32	> 16	> 1
2	Gram Negative	15AUG1997	22A027	ANKARA (KOC)	BLOOD	INTERNAL MEDICINE	OTHER	KLEBSIELLA	PNEUMONIAE	32	> 16	> 16	> 2	> 4	> 4	> 2	> 4	> 4		> 32	<=4	64	> 16	> 1
3	Gram Negative	02APR1998	22A144	ANKARA (KOC)	BLOOD	INTENSIVE CARE	SCEPTOR B.D.	KLEBSIELLA	PNEUMONIAE	16	> 16	> 16	> 2	> 4	> 4		> 4	> 4	> 2	> 16	> 8	> 64	> 16	> 1
4	Gram Negative	08NOV1997	24A013	ISTANBUL	BLOOD	INTERNAL MEDICINE	SCEPTOR B.D.	KLEBSIELLA	PNEUMONIAE	> 32	> 16	> 16	> 2	> 4	> 4	> 2	> 4	> 4		> 32	<=4	<=32	> 16	> 1
5	Gram Negative	08DEC1997	20A288	LONDON	BLOOD	HAEMATOLOGY	OTHER	ESCHERICHIA	COLI	2	> 16	4	> 2	> 4	> 4	> 2	> 4	> 4		> 32	> 8	64	> 16	> 1
6	Gram Negative	06JUL1998	22A168	ANKARA (KOC)	BLOOD	INTENSIVE CARE	API	ESCHERICHIA	COLI	1	> 16	8	> 2	> 4	> 4		> 4	> 4	> 2	> 16	> 8	<=32	> 16	> 1
7	Gram Negative	14APR1998	12A543	UTRECHT	BLOOD	UROLOGY	VITEK	ESCHERICHIA	COLI	4	> 16	1	> 2	0.06	<=0.5		> 4	> 4	> 2	<=8	8	> 64	> 16	> 1
8	Gram Negative	21JUL1998	13A084	WARSAW	BLOOD	PEDIATRICS	OTHER	ESCHERICHIA	COLI	4	> 16	16	> 2	> 4	> 4		4	> 4	> 2	> 16	> 8	64	> 16	1
9	Gram Negative	03MAY1998	15A379	COIMBRA	BLOOD	DERMATOLOGY	VITEK	PSEUDOMONAS	AERUGINOSA	8	> 16	> 16	> 2	> 4	> 4		> 4	> 4	> 2	> 16	> 8	> 64	> 16	> 1

	Amox/clavunalate	Ticarcillin	Ticar/clavunal	Piperacillin	Piper/tazobactam	Cefazolin	Cefuroxime	Cefoxitin	Ceftriaxone	Ceftazidime	Cefepime	Aztreonam	Meropenem	Imipenem
1	> 16	> 128	> 128	> 128	> 64	> 16	> 16	8	> 32	> 16	8	> 16	<=0.06	0.12
2	16	> 128	64	> 128	8	> 16	> 16	8	32	> 16	16	> 16	0.12	0.5
3	> 16	> 128	> 128	> 128	64	> 16	> 16	4	> 32	> 16	4	> 16	0.12	0.25
4	> 16	> 128	> 128	> 128	> 64	> 16	4	4	> 32	> 16	> 16	> 16	0.25	0.5
5	> 16	> 128	> 128	> 128	8	> 16	> 16	> 32	> 32	> 16	4	> 16	0.25	2
6	> 16	> 128	> 128	> 128	16	> 16	> 16	> 32	> 32	> 16	8	> 16	0.25	2
7	> 16	2	<=1	<=1	<=0.5	> 16	0.25	2	> 32	> 16	16	<=0.12	<=0.06	> 8
8	> 16	> 128	> 128	> 128	> 64	> 16	> 16	> 32	32	> 16	> 16	16	4	0.25
9	> 16	> 128	> 128	> 128	> 64	> 16	> 16	> 32	> 32	> 16	> 16	> 16	> 8	> 8
